# Effect of a digital blood pressure coach on hypertension management in primary care practices—a pragmatic, randomised controlled trial

**DOI:** 10.3389/fdgth.2025.1516600

**Published:** 2025-03-07

**Authors:** Christian Beger, Dominik Rüegger, Anna Lenz, Steffen Wagner, Kai Martin Schmidt-Ott, Dirk Volland, Florian P. Limbourg

**Affiliations:** ^1^ Vascular Medicine Research, Department of Nephrology and Hypertension, Hannover Medical School, Hannover, Germany; ^2^ Department of Nephrology and Hypertension, Hannover Medical School, Hannover, Germany; ^3^ Pathmate Technologies GmbH, Mannheim, Germany; ^4^ Department II (Mathematics, Physics and Chemistry), Berliner Hochschule für Technik, Berlin, Germany

**Keywords:** hypertension, home blood pressure monitoring, mHealth, medical apps, primary health care

## Abstract

**Importance:**

Smartphone medical applications (apps) may improve blood pressure (BP) control in the primary care setting in patients with hypertension. However, real-world evidence from primary care is largely lacking.

**Objective:**

To analyse, in primary care practices in Germany, the effect of a smartphone app on systolic BP compared to standard of care.

**Design:**

A pragmatic, non-blinded randomized controlled trial with patients with a diagnosis of hypertension was conducted across 23 general practices in Germany, with a follow-up period of 3 months. Recruitment occurred from January 2022 to May 2023.

**Intervention:**

The intervention group received access to the Manoa app, a smartphone coach integrating guideline-compliant home BP monitoring and lifestyle-coaching. All study participants received standard treatment for arterial hypertension at the discretion of the treating physician.

**Main Outcomes:**

The primary outcome was office systolic BP (oSBP) after 90–150 days in participants with uncontrolled hypertension (oSBP ≥140 mmHg). Secondary outcomes included changes in systolic and diastolic BP, BP control and adherence to home blood pressure monitoring.

**Results:**

A total of 606 participants from 23 general practices were randomized, after data clearance and review, 249 participants were assigned to the control group and 259 to the intervention group for analysis. The mean age (SD) of participants in the intervention group was 55.9 (12.9) years. At baseline, participants with uncontrolled hypertension had a mean oSBP (SD) of 152.6 (14.2) mmHg in the intervention group (*n* = 162) and 152.6 (14.1) mmHg in the control group (*n* = 147). After 120 ± 14 days, oSBP decreased to 137.4 (14.4) mmHg in the intervention group and to 137.7 (14.5) mmHg in the control group, with a between-group mean difference of −0.2 mmHg [95% CI (−3.9,3.5); *P* =.9]. At the follow-up appointment, 69.1% of participants in the intervention group submitted a BP-diary, compared to 36.1% in the control group [OR = 3.95; 95% CI (2.73,5.72); *P* = <0.001].

**Conclusions and Relevance:**

Participants with uncontrolled hypertension randomized to an app in primary care achieved similar decreases in systolic BP but higher adherence to home BP monitoring compared to standard care. In this open-label, pragmatic trial, variability in hypertension management strategies and limited standardization across practices may have confounded the precise evaluation of digital intervention benefits.

**Clinical Trial Registration:**

ClinicalTrials.gov, identifier, (DRKS00027964)

## Introduction

Hypertension (HT) is the most important risk factor for cardiovascular disease and premature death (1). Effective therapy can significantly reduce mortality and morbidity (2). Nonetheless, despite the availability of numerous potent non-pharmacological and pharmacological treatment modalities, the control rate remains insufficient (3).

Home blood pressure monitoring (HBPM) has the potential to improve adherence and blood pressure (BP) control (4). Therefore, various guidelines recommend HBPM for the diagnosis and management of hypertension (5, 6). However, in clinical practice, the consistent implementation of structured guideline-compliant HBPM is often a challenge (7, 8), potentially limiting its clinical utility.

Mobile Health (mHealth) could possibly help to overcome barriers in the diagnosis and treatment of arterial hypertension. For example, smartphone applications (apps) could assist users performing guideline-compliant HBPM and support lifestyle changes such as a better diet or increased physical activity through coaching (9, 10).

Whether these digital interventions also contribute to improved BP-control remains to be definitively clarified. Various stand-alone apps/interventions have shown (slight) improvements in lifestyle or medication adherence, but BP did often not improve consistently (11–13), while more integrated approaches via digital health systems have shown improvements in BP-control in the short and long term (10, 14). Therefore, a crucial question arises regarding the optimal integration of digital interventions into the medical treatment process.

Primary care practices are important for the management of patients with hypertension. HBPM supported by an app could help identify uncontrolled patients in this clinical setting, thereby improving hypertension management and supporting the overcoming of barriers to effective care.

In this study, we aimed to investigate whether a digital coach could support the treatment of patients with uncontrolled hypertension in German general practitioner (GP) offices. The digital coach assists users in performing guideline-compliant HBPM and motivates them to adopt a healthy lifestyle. We assessed its impact on BP management by analysing its effectiveness in lowering blood pressure, improving BP-control, and increasing adherence to HBPM.

## Methods

### General study design

This study is a non-blinded randomised controlled trial aimed to investigate the effect of a smartphone digital application (automated chatbot, Manoa app) on BP-values, BP- control and HBPM compared to standard care in German GP-practices. The Manoa app supports patients to perform guideline-compliant HBPM (4–6) and encourages the implementation of a BP-lowering lifestyle and reliable intake of medication. Recruitment took place from January 17, 2022 to May 5, 2023, allocation ratio was 1:1. Data collection was completed on September 29, 2023.

The study was reviewed and approved by Ethikkommission der Medizinischen Hochschule Hannover, Germany (No. 968_BO_S_2021) and was registered at the German Clinical Trials Register (Registration number: DRKS00027964). All participants provided written informed consent.

### Participants and recruitment

Participants were recruited in 23 GP-practices in Germany (20 in Lower Saxony and 3 in Bavaria). Practice staff assessed potentially eligible participants for inclusion and exclusion criteria. Eligible patients (minimum age 18 years) with pre-diagnosed hypertension had to have sufficient knowledge of German and an email address. Furthermore, access to the internet and to a smartphone was required. Patients who had already used the app in the past were excluded from participating in the study. Further exclusion criteria were pregnancy and severe (uncorrectable) visual impairment, which precluded the use of the app (Supplementary Table S1). Eligible participants were identified by employees of the medical practices (doctors or medical assistants). If the requirements for admission were met, written consent was obtained after written and verbal study information.

### Randomization

After enrollment and consent participants were assigned to study groups (digital coach + standard care vs. standard care alone) by randomization. Randomization was based on a centralized computer-generated assignment sequence. A block-randomized list was created for each study center using the R package “blockrand,” (15) with each block containing 6 participant IDs. The sequence was generated randomly by the study management using the package randomizeR (16), which is implemented in the statistics programme R (17).

### Intervention

All study participants received standard treatment for arterial hypertension as per discretion of the treating physician. The participants in the intervention groups also received access to the Manoa app. The app and its features were recently described (18). In summary, the app interacts with patients via a chatbot and provides information on blood pressure, correct BP-measurement and healthy lifestyle. The core element is the HBPM module: Patients are instructed to measure their BP twice a day (morning and evening) for 6 days and to document their BP-values in the app. Based on the average BP-value, the patient is advised to make an appointment with a doctor if their BP is elevated (≥ 135 mmHg). After completion of the first measurement week, the patients of the intervention group (IG) are repeatedly asked to perform a new measurement week at intervals of 4 weeks.

In addition, coaching programs are provided in the areas of exercise, nutrition and relaxation. The user receives structured information and is encouraged to define individual behavioral goals. The chatbot supports practical implementation through daily reminders and informs about strategies for effective self-management. The recommendations are based on guidelines (5, 6) and the individual user data (e.g., age, height, weight).

### Measurements and outcomes

The office BP was measured at baseline and after 120 ± 14 days as part of a routine examination by the practice staff, following best practices. In addition, relevant basic information (e.g., year of birth, weight, height, gender, relevant concomitant diseases, duration of hypertension diagnosis, antihypertensive medication) were documented upon enrolment. As part of the follow-up, antihypertensive medication (type, number, dose), adherence to HBPM and (if applicable) cardiovascular events were documented. If available, an ambulatory blood pressure measurement (ABPM) was also performed.

The primary endpoint was defined as the reduction in office systolic BP (oSBP) at follow-up compared to baseline in patients with uncontrolled hypertension (≥140/90 BP; between group difference). Secondary endpoints included BP-control rate and BP-reduction at follow up in all participants with pre-diagnosed hypertension. In addition, the effect of the app on adherence to HBPM and patient behaviour with regard to check-ups and medication was analysed. Subgroups for further analyses were defined based on relevant characteristics, e.g., age, gender, BP-categories, comorbidities, duration of initial hypertension diagnosis (Supplementary Table S2). Defined daily doses of medications were calculated according to the 2018 official ATC index with DDD information for Germany (19).

### Sample size

We conducted a power analysis to determine the sample size for detecting a clinically significant difference in oSBP among participants diagnosed with uncontrolled hypertension (oSBP ≥ 140 mmHg). This was grounded in the outcomes of a 2021 meta-analysis (2) encompassing 48 randomized trials, which demonstrated that a reduction in systolic BP by 5 mmHg, could lead to a 10% decrease in the risk for cardiovascular events, such as heart attacks or strokes, over a subsequent five-year period. In alignment with comparable studies (14, 20), the sample size was designed to detect a difference of 5 mmHg (SD 15 mmHg) in the reduction in systolic BP between the intervention group and the control group (difference-in-differences).

To achieve 80% power (β = 0.2) at a significance level of 0.05, our calculations indicated a required sample size of 286 participants with uncontrolled hypertension. To account for an anticipated dropout rate of 10%, we aimed to recruit 318 patients with uncontrolled systolic BP.

The current study recruited participants diagnosed with arterial hypertension, including participants with controlled and uncontrolled hypertension. Based on two national health examination surveys (3), we estimated that approximately one-third (33%) of the participants would have uncontrolled BP at baseline. To achieve this target, we aimed to recruit a total of 954 participants, ensuring that 318 participants would have uncontrolled BP at the baseline measurement. Since the actual prevalence of uncontrolled hypertension within our study cohort was higher than initially anticipated, the target number of participants for the primary analysis (286 individuals with elevated BP) was achieved after including 525 participants with complete data sets. The higher prevalence of uncontrolled hypertension among our study population led to an early completion of participant enrolment and 317 included individuals with elevated systolic BP in the current study.

### Statistics

The analyses were performed with the open-source statistical software R (17). All analyses were conducted as intention-to-treat analyses using data from participants with complete data sets. Characteristics of participants were summarised as numbers and percentage for categorical variables and mean and SD for continuous variables. Continuous variables such as change in BP- level were analysed with a *t*-test. Changes in discrete variables over time such as hypertension control were analysed using a logistic regression considering an additional random effect for the study participants. The mixed logistic regression with random-intercepts for individuals applied to model the categorical dichotomous outcome variable controlled BP (yes/no) considered group membership (treatment/control) and time of data collection (begin/end of study) as well as the interaction of both measures resulting in the R (21) specific model formulation lme4::glmer[controlled∼is_treatment * time + (1 | ID), family = binomial(), [..]].

Effect sizes are given as Cohen's d for continuous variables and as odds ratio for effects on discrete outcomes (22). All tests were performed two-sided. All analyses were performed for original assigned groups.

## Results

### Participants

**Figure 1:**
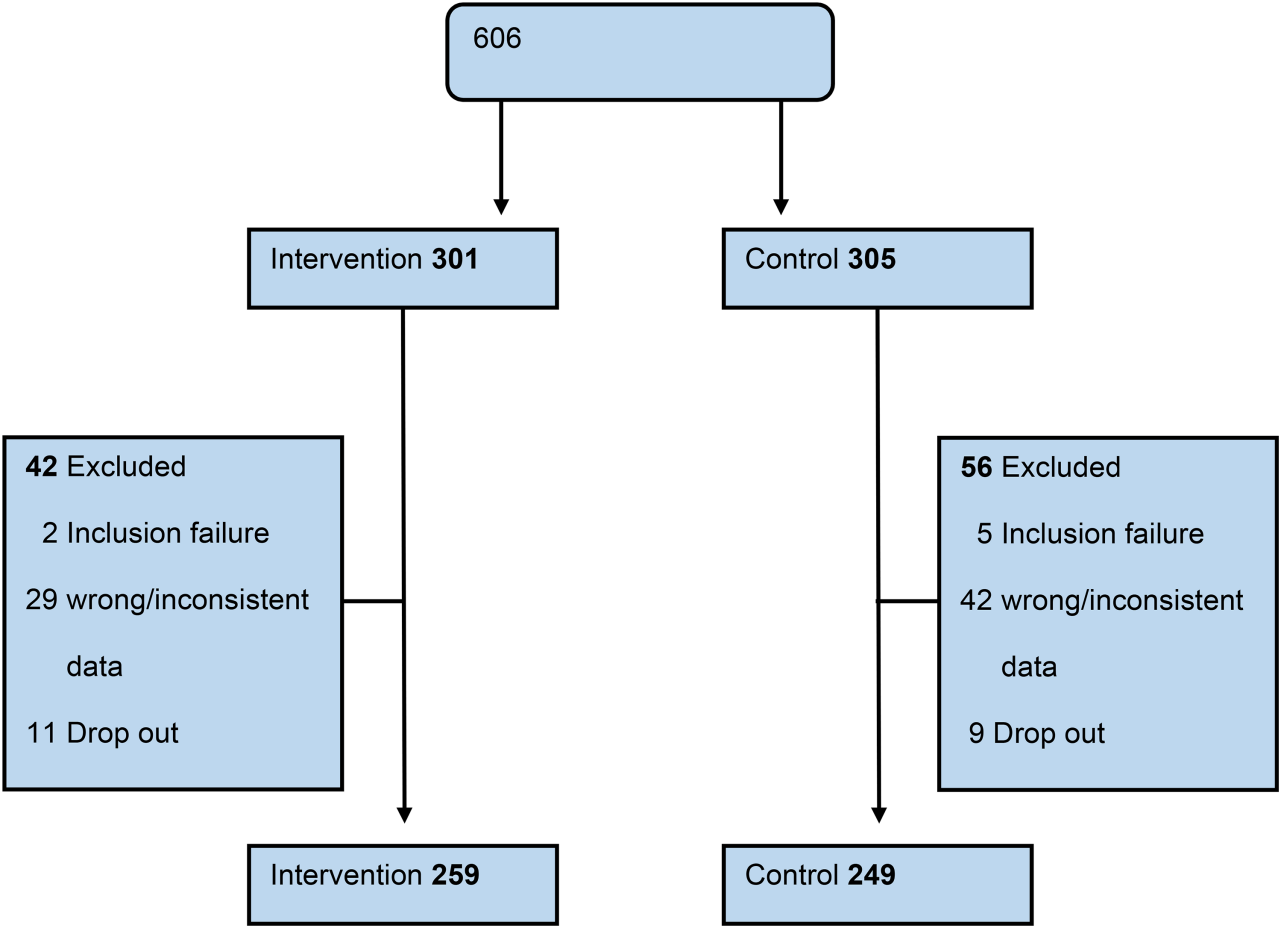
Patient flow diagram.

Overall, 606 participants were randomised from 23 GP-practices to achieve the predefined number of uncontrolled patients. This number was smaller than anticipated due to a higher than expected prevalence of uncontrolled hypertension in this population. 301 were assigned to the intervention group and 305 to the control group. In 12.9% the data sets were incomplete or inconsistent, in 1.2% a further review revealed that the inclusion criteria were not met. 86.0% of the intervention group were included in the final analysis (81.6% control, Figure 1).

### Baseline characteristics

The mean age (SD) of the participants in the intervention group was 55.9 (12.9) years and 40.9% of them were female. In the control group, the average age was 55.5 (13.8) years and 43% of the participants were female. 90.7% of the participants in the intervention group received antihypertensive medication (control: 88.4%). Overall, the two groups exhibited comparable baseline characteristics (Table 1).

**Table 1:** Baseline characteristics.

Characteristics	Participants, *n* (%)	
	Intervention:	Control:
	Coaching App	Standard care
	(*n* = 259)	(*n* = 249)
Age, years	55.9 ± 12.9	55.5 ± 13.8
Female (%)	106 (40.9)	107 (43.0)
Body mass index	29.2 ± 5.2	30.3 ± 9.3
Office SBP, mmHg	142.7 ± 17.9	142.3 ± 17.3
Office DBP, mmHg	87.5 ± 11.8	86.7 ± 10.5
Office BP controlled (%)	82 (31.7)	82 (32.9)
Office SBP uncontrolled (%)	162 (62.5)	147 (59.0)
Office DBP uncontrolled (%)	124 (47.9)	112 (45.0)
Antihypertensive medication (%)	235 (90.7)	220 (88.4)
Defined daily dose	2.6 ± 1.9	2.6 ± 2.1
Comorbidities (%)		
- Diabetes	23 (8.9)	32 (12.9)
- Chronic kidney disease	4 (1.5)	8 (3.2)
- Coronary heart disease	22 (8.5)	20 (8.0)
- Chronic obstructive pulmonary disease	1 (0.4)	5 (2.0)

Baseline characteristics of all participants. Variables expressed as number and percentage. Age, Body mass index, office systolic and diastolic BP values, antihypertensive agents and defined daily doses are expressed as mean ± SD. Controlled office BP: systolic BP <140 mm Hg and diastolic BP <90 mm Hg.

### Change in blood pressure and blood pressure control

At baseline, the mean systolic office BP (SD) was 142.7 (17.9) mm Hg in the intervention group and 142.3 (17.3) mm Hg in the control group. Initially, 31.7% of intervention participants had BP within the controlled range, compared to 32.9% in the control group (Table 1).

In participants of the intervention group with uncontrolled hypertension, the initial oSBP (SD) was 152.6 (14.2), compared to 152.6 (14.1) in the control group. After 120 ± 14 days, those using the digital coach experienced a decrease in oSBP (SD) to 137.4 (14.4), similar to the control group's decrease to 137.7 (14.5). The between-group mean difference was −0.2 mmHg [95% CI (−3.9,3.5); *P* = .9; Table 2].

**Table 2:** Change in blood pressure and BP-control in participants with uncontrolled hypertension.

BP	Coaching app—intervention (*n* = 162)			Standard care—control (*n* = 147)			Effect-size	Statistic	95% CI	*P*
	Baseline	Follow up	Change	Baseline	Follow up	Change				
oSBP (SD)^a^	152.6 ± 14.2	137.4 ± 14.4	−15.2 ± 16.4	152.6 ± 14.1	137.7 ± 14.5	−15.0 ± 16.7	−0.01	t = 0.1	−3.94–3.48	.9
oSBP control (%)	0	54.9	54.9	0	52.4	52.4	1.11 OR	Z = 0.5	0.71–1.73	.65

Data for patients with uncontrolled systolic hypertension (≥ 140 mmHg). OBP was measured at baseline and after 120 ± 14 days. Effect size of oSBP control reported as odds ratio (OR). oSBP: office systolic BP, oSBP control: oSBP <140 mm Hg.

^a^ Primary outcome.

Among all intervention group participants, the mean oSBP (SD) decreased to 134.8 (14.2) mm Hg, reflecting an average reduction of 7.9 (18.2) mm Hg from baseline. In contrast, the control group's mean BP (SD) decreased to 135.8 (15.5) mm Hg, a reduction of 6.5 (19.8) mm Hg from baseline. The between-group mean difference was −1.4 mm Hg [95%CI (−4.7,1.9); *P* = .4]. The intervention was associated with an increase in the BP-control rate from 31.7% to 54.4%; which was comparable to the effect observed in the control group [OR = 1.11; 95% CI (0.63,1.97); *P* = 0.71; Table 3]. No substantial changes in the defined daily dose (DDD) were observed in either the control or intervention group (Supplementary Table S5). Interim visits between the baseline and follow-up visits were negligible (Intervention: *n* = 0.3 ± 0.7; Control: *n* = 0.1 ± 0.3; data not shown).

**Table 3:** Change in blood pressure and BP-control in all participants.

BP	Coaching app—intervention (*n* = 259)			Standard care—control (*n* = 249)			Effect-	Statistic	95% CI	*P*
	Baseline	Follow up	Change	Baseline	Follow up	Change	size			
oSBP (SD)	142.7 ± 17.9	134.8 ± 14.2	−7.9 ± 18.2	142.3 ± 17.3	135.8 ± 15.5	−6.5 ± 19.8	−0.07	t = −0.8	−4.7–1.9	.40
oDBP (SD)	87.5 ± 11.8	81.9 ± 9.3	−5.6 ± 13.0	86.7 ± 10.5	81.5 ± 9.2	−5.1 ± 11.4	−0.04	t = −0.4	−2.6–1.6	.67
oSBP control (%)	37.5	63.3	25.9	41.0	59.4	18.5	1.44 OR	z = 1.3	0.83–2.49	.19
oDBP control (%)	52.1	76.1	23.9	55.0	78.3	23.3	1.01 OR	z = 0.0	0.54–1.89	.98
OBP control (%)	31.7	54.4	22.8	32.9	53.8	20.9	1.11 OR	z = 0.4	0.63–1.97	.71

Data for all participants. BP was measured at baseline and after 120 ± 14 days in the practice. oSBP control: systolic BP <140 mm Hg. oDBP control:<90 mm Hg. Controlled BP: oSBP < 140 mm Hg and office DBP <90 mm Hg. OBP: Office blood pressure, OR: odds ratio.

### Change in blood pressure and blood pressure control in different subgroups

The effect of the BP coaching app on BP was analysed in pre-specified subgroups (Table 4). The mean between-group difference in systolic BP between female participants in the intervention and control group after 120 ± 14 days was not significantly different—with a decrease of −0.1 mm Hg [95% CI (−5.2, 5.1); *P* = .99] observed in the intervention group compared to the control group. Among male participants, the between-group difference in systolic BP indicated a mean decrease of 2.4 mm Hg [95% CI (−6.8,1.9); *P* = .27] in the intervention group compared to the control group. The mean BP difference for older participants (age ≥60 years) was −3.0 mm Hg [95% CI, (−8.6,2.7); *P* = .30]. For younger participants the corresponding BP difference was −0.1 mm Hg [95% CI, (−4.1, 3.9); *P* = .95]. In addition, the mean BP difference for obese subjects with BMI ≥30 kg/m^2^ was −1.8 mm Hg [95% CI, (−7.3,3.7); *P* = .53].

**Table 4:** Analysis of change in blood pressure in different subgroups (between group difference).

Subgroup	Coaching app—intervention				Standard care—control				Effect-size	Statistic	95% CI	*P*
	*n*	Baseline	Follow up	Change	*n*	Baseline	Follow up	Change				
Female	106	141.5 ± 20.1	133.5 ± 13.3	−8.0 ± 19.2	107	143.2 ± 17.6	135.3 ± 15.5	−7.9 ± 19.0	−0.00	*t* = −0.0	−5.2–5.1	.99
Male	153	143.6 ± 16.2	135.8 ± 14.8	−7.9 ± 17.5	142	141.6 ± 17.1	136.2 ± 15.5	−5.4 ± 20.4	−0.13	*t* = −1.1	−6.8–1.9	.27
Age <60y	156	142.0 ± 16.1	133.4 ± 11.6	−8.6 ± 17.4	142	143.7 ± 16.0	135.3 ± 13.4	−8.4 ± 17.9	−0.01	*t* = −0.1	−4.2–3.9	.95
Age ≥60y	103	143.9 ± 20.3	137.0 ± 17.3	−6.9 ± 19.3	107	140.5 ± 18.8	136.6 ± 17.9	−3.9 ± 22.0	−0.14	*t* = −1.0	−8.6–2.7	.30
BMI <30 kg/m^2^	155	142.8 ± 18.6	134.9 ± 13.0	−7.9 ± 18.6	142	142.1 ± 16.7	135.3 ± 15.0	−6.8 ± 17.6	−0.06	*t* = −0.5	−5.3–3.0	.59
BMI ≥30 kg/m^2^	104	142.7 ± 16.9	134.8 ± 16.0	−7.9 ± 17.5	107	142.7 ± 18.1	136.5 ± 16.1	−6.1 ± 22.6	−0.09	*t* = −0.6	−7.3–2.6	.53
BP controlled	82	125.0 ± 9.0	129.0 ± 12.3	4.0 ± 14.2	82	126.8 ± 8.9	133.3 ± 17.5	6.5 ± 18.5	−0.15	*t* = −1.0	−7.5–2.6	.34
BP uncontrolled	177	151.0 ± 14.7	137.6 ± 14.3	−13.4 ± 17.2	167	149.9 ± 15.2	137.1 ± 14.3	−12.9 ± 17.2	−0.03	*t* = −0.3	−4.2–3.1	.76

OBP was measured at baseline and after 120 ± 14 days. Controlled OBP: oSBP < 140 mm Hg and oDBP <90 mm Hg. OBP: Office blood pressure.

### Effect of the coaching app on home blood pressure monitoring

A core feature of the app is the HBPM module, which assists users in performing HBPM according to guideline-compliant measurement protocols. At follow-up 69.1% of participants in the intervention group submitted a BP-diary, compared to 36.1% in the control group [OR = 3.95; 95% CI (2.73,5.72); *P* = <0.001]. Additionally, 67.2% of app users met the guideline requirements, while only 32.5% of the control group achieved guideline compliance [OR = 4.25; 95% CI (2.93,6.15); *P* = <0.001]. The BP-diary was subsequently used for therapy assessment in 68.3% of the intervention group, vs. 32.1% in the control group [OR = 4.56; 95% CI (3.14,6.62); *P* = <0.001, Table 5].

**Table 5:** Effect of a coaching app on home blood pressure monitoring.

Outcome	Coaching app—intervention		Standard care—control		Effect-size	Statistic	*P*
	*n*	%	*n*	%			
BP-diary submitted	259	69.1	249	36.1	3.95	Z = 7.30	<0.001
Guideline-compliant BP- diary submitted	259	67.2	249	32.5	4.25	Z = 7.64	<0.001
BP-diary is used to assess therapy	259	68.3	249	32.1	4.56	Z = 7.97	<0.001
Guideline-compliant BP diary + use for therapy assessment	259	66.4	249	30.9	4.42	Z = 7.82	<0.001

Data for all participants.

## Discussion

In this pragmatic, primary care practice-based randomized trial, the use of a BP coaching app compared to standard of care did not result in better systolic BP reduction or better hypertension control among participants with uncontrolled hypertension. In both groups, a strong office BP reduction was observed between baseline and follow-up BP. Participants in the intervention group were significantly more likely to use a guideline-adherent BP- diary. Additionally, physicians used HBPM data for management more often in the app group than in the control group.

The available evidence concerning the effect of mHealth on BP remains limited and the reported study results are heterogeneous (23). In the Smart Hypertension Control Study, a tracking app was compared with a coaching app designed to enhance patients’ health literacy and self-management skills. In addition to structured HBPM, various aspects of lifestyle management were addressed. After 6 months, no significant difference in systolic BP was observed between the groups (13). However, in another study encompassing 480 participants, a significantly stronger decrease in BP was recorded after 6 months among those who used the “YanFu” app compared to the control group. The control group solely documented their daily BP on paper, while the app not only reminded users to measure their BP but also enabled remote consultations with doctors (24).

In principle, the various apps differ significantly in design and approach, which limits direct comparisons. Therefore, when considering different studies, the observed effect on BP-reduction may depend on the degree of integration of the app into the treatment process. A recent study examined an app connected to a practice management platform, enabling direct communication between patients and doctors. This app provided medication recommendations based on HBPM results and physicians’ instructions, significantly reducing BP and improving control (25). Similarly, the “YanFu” app facilitated data monitoring by physicians and allowed for remote consultations (24).

In our trial, treatment decisions based on HBPM were not formally integrated but were left to the discretion of the physician. Also, the digital coach did not enable direct interaction between doctors and patients. For uncontrolled hypertension during HBPM, the coach advised participants to schedule a doctor's appointment. However, participants mainly attended the scheduled final appointment after three months, bringing their BP-diaries, with few additional appointments in between. This reluctance could be attributed to the pre-scheduled three-month follow-up appointment, possibly leading participants to perceive an earlier appointment as unnecessary.

Similarly, in the Smart Hypertension Control Study, there was no increase in medical consultations or medication adjustments, as consultations were only suggested for consistently abnormal BP readings (13). A stronger integration of the digital coach examined in this study into the treatment process might enhance the app's effectiveness. The study design lacked an additional measurement point. Specifically, a follow-up assessment at six months after all participants had visited their physician and presented their HBPM readings would have been beneficial. This additional data point could have offered more comprehensive insights into the long-term effects of the app on BP-management and the impact of facilitated monitoring through HBPM readings. Furthermore, other studies have shown that firm integration of HBPM into the study protocol can enhance the intervention's effectiveness. In the HOME BP trial, self-monitoring of BP with a digital intervention resulted in better reduction and control of systolic BP after one year (10). Another study demonstrated that self-monitoring used by general practitioners to adjust antihypertensive medication in patients with uncontrolled hypertension results in significantly lower BP compared to adjustments guided by office measurements^18^.

As this study was designed as a pragmatic, primary care-based trial, physicians were not specifically advised to integrate the digital coach/HBPM in care. Thus, hypertension management was potentially not solely based on HBPM; rather, the digital coach served as an additional potential component. It remains unclear how extensively it was utilized for management.

Additionally, this study observed a significant decrease in systolic BP within the control group [oSBP (SD) −6.5 mmHg (19.8)], which is comparable with the findings from the Smart Hypertension Control Study (approximately −6.8 mmHg) (13). In another randomized controlled study, the effect of an interactive smartphone app was investigated in hypertensive patients who had not yet received any antihypertensive medication. Participants were recruited by hypertension centers and randomized to conventional lifestyle-coaching or app-based lifestyle coaching. The app group showed a significantly greater reduction in average 24 h systolic BP (−2.4 mmHg) and HBPM-based BP-measurements, while the effects in the control group were significantly smaller (10). Compared to this study, our study included patients with existing medication. While the net BP-lowering effect in the app groups was similar, our study found a significant BP reduction in the control group as well. This may indicate an awareness effect among participating physicians for uncontrolled patients, leading to medication adjustments before inclusion.

The core element of the digital coach is the HBPM module. Structured HBPM is recommended by guidelines for the diagnosis and management of hypertension (5), but its implementation is often challenging in clinical practice (7, 8). It is therefore noteworthy that participants in the intervention group completed significantly more guideline-compliant HBPM measurements. However, this did not have an impact on BP-control during the short observation period. Nevertheless, it is known that structured HBPM can improve adherence, a critical factor for long-term BP- control (26, 27). Similar considerations apply to non-pharmacological interventions (e.g., healthy diet, physical activity) which are recommended by guidelines. Behavioural changes require time—thus, it is unlikely to expect a measurable effect within a 12-week study period. Longer study durations or real-world data would be necessary to thoroughly examine the long-term effects of the HBPM-module and non-pharmacological interventions on adherence and BP-control.

This study revealed no positive effect of a digital coaching app on BP reduction or control compared to standard care. However, it offers valuable additional insights. Unlike many previous studies conducted in specialized centers, this study was performed in a primary care setting, which highlights potential challenges associated with this approach. Standardizing clinical processes in primary care practices is only partially achievable, reflecting the reality of clinical practice. Additionally, HBPM is just one aspect of hypertension management, complicating the precise evaluation of a digital intervention's benefits. Importantly, the ability for low-threshold, direct interaction between medical professionals and patients appears crucial for the optimal use of digital applications in both studies and everyday clinical practice.

### Strengths and limitations

This study has limitations. Due to the design (digital coach + standard care vs. standard care alone), blinding was not feasible. However, randomization was conducted using a randomly generated list. Furthermore, this study was designed as a pragmatic, primary care-based trial, conducted not in study centers but in the participating practices. Both groups received standard of care, which was not precisely predefined and may vary between individual practices. This heterogeneity aligns with clinical practice. Since the app is also intended for use in primary care, this limited level of standardization seems reasonable and potentially beneficial. Furthermore the pragmatic study design limited the amount of data which could be collected. For example, qualitative feedback from participants would have been beneficial to better understand user behavior, adherence and thereby differences between the control and intervention group. ABPM was not analysed systematically. This could have been useful for a more comprehensive evaluation of BP-control. The study duration was approximately 120 days, which is shorter than most other studies (24, 25). The duration of the study might have been too short to demonstrate the effects of the digital coach, HBPM, or lifestyle interventions on BP. Generally, the results of this study may not be directly applicable to other mHealth interventions.

## Data Availability

The raw data supporting the conclusions of this article will be made available by the authors, without undue reservation.
