# Supplementary material for: Effect of a digital blood pressure coach on hypertension management in primary care practices—a pragmatic, randomised controlled trial
Source: Front Digit Health. 2025 Mar 7;7:1516600. doi: 10.3389/fdgth.2025.1516600 (PMC11925922; doi:10.3389/fdgth.2025.1516600)
Supplement: Supplementary file 1 [file Datasheet1.pdf]

Supplementary Material

Effect of a digital blood pressure coach on hypertension management in primary care practices - a pragmatic, randomised controlled trial

Christian BEGER[a,b], Dominik RÜEGGER[c], Anna LENZ[c], Steffen WAGNER[d], Kai Martin SCHMIDT-OTT[b], Dirk VOLLAND[c], Florian P. LIMBOURG[a, b]

[a] Vascular Medicine Research, Dept. of Nephrology and Hypertension, Hannover Medical School, Hannover, Germany

[b] Department of Nephrology and Hypertension, Hannover Medical School, Hannover, Germany

[c] Pathmate Technologies GmbH, Mannheim, Germany

[d] Department II (Mathematics, Physics and Chemistry), Berliner Hochschule für Technik, Berlin, Germany.

TABLE S1. Inclusion and Exclusion criteria

Inclusion criteria
- Minimum age of 18 years
- Diagnosis of hypertension (ICD 10: I10)
- Sufficient knowledge of German
- Existing E-Mail address
- Internet access
- Smartphone ownership
**Exclusion criteria**
- Severe (uncorrectable) visual impairment that may hinder the use of the app
- Pregnancy

TABLE S2. Trial Outcomes

Primary outcome
Between group difference (control vs. intervention group) in office SBP from time t0 to time t1 (routine examination after 90–150 days) in participants with uncontrolled hypertension.
**Secondary outcomes**
1. Between group difference (control vs. intervention group) in office SBP from time t0 to time t1 (routine examination after 90–150 days) in all participants.<br>2. Between group difference (control vs. intervention group) in office DBP from time t0 to time t1 (routine examination after 90–150 days) in participants with uncontrolled hypertension.<br>3. Between group difference (control vs. intervention group) in office DBP from time t0 to time t1 (routine examination after 90–150 days) in all participants.<br>4. Between group difference (control vs. intervention group) in oSBP-control rate from time t0 to time t1 (routine examination after 90–150 days) in all participants.<br>5. Between group difference (control vs. intervention group) in oDBP-control rate from time t0 to time t1 (routine examination after 90–150 days) in all participants.<br>6. Difference in the proportion of patients in the IG and CG with a valid blood pressure diary.

Time t0= baseline, time t1= after 90–150 days.

TABLE S3. Change in blood pressure and BP-control in participants with uncontrolled diastolic hypertension

	Coaching app - Intervention (n=162)			Standard care - Control (n=147)			Effect-size			
	Baseline	Follow up	Change	Baseline	Follow up	Change		Statistic	95% CI	P
Office DBP (SD)	96.3±10.4	85.0±9.3	-11.3±14.4	95.7±6.5	84.6±8.6	-11.1±10.5	-0.20	t=0.1	-3.42-3.02	.9
Office DBP control (%)	0	61.3	61.3	0	67	67	0.78 OR	Z=0.9	0.46-1.33	.36

Data for participants with uncontrolled diastolic hypertension (≥ 90mmHg). OBP was measured at baseline and after 120 ± 14 days. Office DBP control: office DBP<90 mm Hg. OBP: Office blood pressure, DBP: diastolic BP, OR: odds ratio.

TABLE S4. Analysis of change in blood pressure control in various subgroups

	Coaching app - Intervention				Standard care - Control				Effect-			
	n	Baseline	Follow up	Change	n	Baseline	Follow up	Change	size	Statistic	95% CI	*P*
Female	106	39.6	57.5	17.9	107	33.6	57.9	24.3	0.71	z=-0.8	0.30-1.71	.45
Male	153	26.1	52.3	26.1	142	32.4	50.7	18.3	1.57	z=1.2	0.73-3.38	.25
Age <60y	156	31.4	55.8	24.4	142	28.2	50.7	22.5	1.07	z=0.2	0.51-2.25	.86
Age ≥60y	103	32.0	52.4	20.4	107	39.3	57.9	18.7	1.13	z=0.3	0.46-2.80	.79
BMI <30kg/m^2	155	31.6	50.3	18.7	142	34.5	57.7	23.2	0.79	z=-0.6	0.36-1.73	.56
BMI ≥30 kg/m^2	104	31.7	60.6	28.8	107	30.8	48.6	17.8	1.66	z=1.2	0.71-3.91	.24
BP controlled	82	100.0	75.6	-24.4	82	100.0	69.5	-30.5	1.36	z=0.9	0.68-2.71	.38
BP uncontrolled	177	0	44.6	44.6	167	0	46.1	46.1	0.94	z=-0.3	0.62-1.44	.76

OBP was measured at baseline and after 120 ± 14 days. Controlled OBP: oSBP < 140 mm Hg and office DBP< 90 mm Hg. OBP: Office blood pressure

TABLE S5. Change in Defined Daily Dose (DDD)

	Coaching app - Intervention				Standard care - Control			
	n	Baseline	Follow up	Change	n	Baseline	Follow up	Change
DDD mean (SD)	258	2.6 (1.9)	2.8 (2.0)	0.2 (1.1)	245	2.6 (2.1)	2.6 (2.0)	0.0 (1.2)
DDD median (IQR)	258	2.0 (0-10.9)	2.1 (0-8.7)	0.0 (-4.5-5)	245	2.0 (0-10.2)	2.0 (0.-11.4)	0.0 (-5.8-5.0)

DDD: Defined daily dose, SD: standard deviation, IQR: interquartile range.

TABLE S6. Characteristics of participants and excluded individuals at baseline

Characteristics	Participants, n (%)			
	Intervention: Coaching App		Control: Standard care	
	Participants (n=259)	Excluded (n=42)	Participants (n=249)	Excluded (n=55)
Age, years	55.9±12.9	55.4±16.1	55.5±13.8	50.1±15.4 (n=53)
Female (%)	106 (40.9)	16 (40, n=40)	107 (43.0)	21 (40.4, n=50)
Body mass index	29.2±5.2	28.3±5.6	30.3±9.3	31.2±8.6
Office SBP, mmHg	142.7±17.9	145.9±15.5 (n=41)	142.3±17.3	140.7±19.0 (n=54)
Office DBP, mmHg	87.5±11.8	88.3±11.2 (n=41)	86.7±10.5	87.2±10.6 (n=54)
Office BP controlled (%)	82 (31.7)	9 (21.9, n=41)	82 (32.9)	20 (37.0, n=54)
Antihypertensive medication (%)	235 (90.7)	34 (80.9)	220 (88.4)	45 (83.3, n=54)
Defined daily dose	2.6±1.9	2.5±2.2	2.6±2.1	2.4±2.1 (n=47)

Characteristics of all participants. Variables expressed as number and percentage. Age, Body mass index, office systolic and diastolic BP values, antihypertensive agents and defined daily doses are expressed as mean ± SD. Controlled office BP: systolic BP < 140 mm Hg and diastolic BP < 90 mm Hg. Due to incomplete data for the excluded participants, the n number varies.
